# [6]-Shogaol Inhibits *α*-MSH-Induced Melanogenesis through the Acceleration of ERK and PI3K/Akt-Mediated MITF Degradation

**DOI:** 10.1155/2014/842569

**Published:** 2014-06-19

**Authors:** Huey-Chun Huang, Shu-Jen Chang, Chia-Yin Wu, Hui-Ju Ke, Tsong-Min Chang

**Affiliations:** ^1^Department of Medical Laboratory Science and Biotechnology, College of Health Care, China Medical University, Taichung, Taiwan; ^2^School of Pharmacy, China Medical University, Taichung, Taiwan; ^3^Department of Applied Cosmetology and Master Program of Cosmetic Sciences, Hung Kuang University, Taichung, Taiwan

## Abstract

[6]-Shogaol is the main biologically active component of ginger. Previous reports showed that [6]-shogaol has several pharmacological characteristics, such as antioxidative, anti-inflammatory, antimicrobial, and anticarcinogenic properties. However, the effects of [6]-shogaol on melanogenesis remain to be elucidated. The study aimed to evaluate the potential skin whitening mechanisms of [6]-shogaol. The effects of [6]-shogaol on cell viability, melanin content, tyrosinase activity, and the expression of the tyrosinase and microphthalmia-associated transcription factor (MITF) were measured. The results revealed that [6]-shogaol effectively suppresses tyrosinase activity and the amount of melanin and that those effects are more pronounced than those of arbutin. It was also found that [6]-shogaol decreased the protein expression levels of tyrosinase-related protein 1 (TRP-1) and microphthalmia-associated transcriptional factor (MITF). In addition, the MITF mRNA levels were also effectively decreased in the presence of 20 *μ*M [6]-shogaol. The degradation of MITF protein was inhibited by the MEK 1-inhibitor (U0126) or phosphatidylinositol-3-kinase inhibitor (PI3K inhibitor) (LY294002). Further immunofluorescence staining assay implied the involvement of the proteasome in the downregulation of MITF by [6]-shogaol. Our confocal assay results also confirmed that [6]-shogaol inhibited *α*-melanocyte stimulating hormone- (*α*-MSH-) induced melanogenesis through the acceleration of extracellular responsive kinase (ERK) and phosphatidylinositol-3-kinase- (PI3K/Akt-) mediated MITF degradation.

## 1. Introduction

Edible phytochemicals are now considered to be safe, inexpensive, readily acceptable, and promising depigmentation agents. Ginger rhizome (*Zingiber officinale *Roscoe) is one of the oldest herbs that is officially listed in the traditional Chinese Pharmacopoeia. [6]-Shogaol, the major shogaol in ginger rhizomes [1, 2], has been found to possess many interesting pharmacological and physiological activities, such as antiproliferation [3] and antioxidant activities [4]. As a traditional “antioxidant” agent, the antimelanogenesis activity of [6]-shogaol has not been well documented. Previous studies have supported the potential of [6]-shogaol as a melanogenesis inhibitor agent. In this study, we explored the possible mechanisms of [6]-shogaol on melanin synthesis.

The biosynthesis of melanin is a complicated process involving many factors. Alpha-melanocyte stimulating hormone (*α*-MSH) is the most important melanocyte-stimulating hormone in stimulating melanogenesis. *α*-MSH binds to melanocortin 1 receptor (MC1R), which induces the activation of adenylyl cyclase, followed by cAMP production [5, 6]. cAMP leads to phosphorylation of CREB transcription factors, which in turn stimulate microphthalmia transcription factor (MITF) promoter activation. MITF directly binds to the promoter regions of melanin production genes and positively regulates their transcription; these genes include at least three melanocyte-specific enzymes: tyrosinase, tyrosinase-related protein 1 (TRP1), and tyrosinase-related protein 2 (TRP2) [7–9]. Tyrosinase catalyzes the production of melanin and other pigments from tyrosine by oxidation [10]. TRPs may be important in the regulation of melanogenesis or in the assembly of the melanogenic apparatus [11, 12]. *α*-MSH induces MITF expression by activating several signaling pathways. The activation of ERK phosphorylates MITF, which is followed by MITF ubiquitination and degradation. In addition, p38 MAPK has recently been shown to be involved in UVR-induced melanogenesis and leads to the activation of MITF expression and consequently increased tyrosinase expression [13]. Furthermore, the MITF gene is transcriptionally upregulated by phosphatidylinositol-3-kinase (PI3K) signaling in melanocytes [14] and is thus tightly regulated in a signal-dependent fashion; this in turn affects MITF expression and the regulation of pigmentation. In addition to transcriptional regulation, MITF is also subject to various posttranslational modifications, particularly phosphorylation by ERK [15], ribosomal S6 kinase (RSK) [16], glycogen synthase kinase-3*β* (GSK3*β*) [17], and p38 [18], thus targeting MITF for degradation.

It has been reported that some antioxidants, such as kojic acid [19] and ascorbic acid derivatives [20], play an important role in the inhibition of melanogenesis, which implies the close association between antioxidative mechanisms and the downregulation of hyperpigmentation. Recently, the antimelanogenic effects of [6]- and [8]-gingerol have been reported [21, 22]. However, the effects of other components of ginger rhizome on the melanogenesis signaling pathway have not been investigated. In this study, we examined the effect of [6]-shogaol on *α*-MSH-induced melanogenesis in mouse melanoma B16F10 cells. In particular, we analyzed changes in the ERK signaling pathway and the associated MITF regulation.

## 2. Materials and Methods

### 2.1. Materials

**Figure 1:**
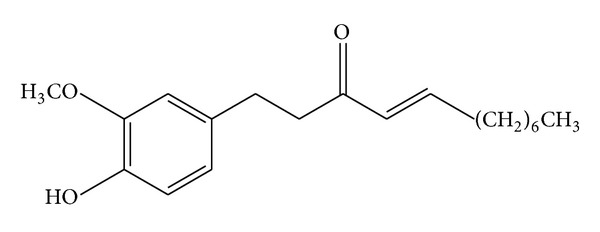
Chemical structure of [6]-shogaol (C_17_H_24_O_3_).

[6]-Shogaol (Figure 1) and kojic acid were purchased from Wako Pure Chemical Industries (Osaka, Japan). All other chemicals and solvents were obtained from Sigma-Aldrich (Saint Louis, MO).

### 2.2. Cell Culture

B16F10 cells (ATCC CRL-6475; BCRC60031) were cultured in Dulbecco's modification of Eagle's medium (DMEM) with 10% fetal bovine serum (FBS; Gibco, Langley, OK, USA) and penicillin/streptomycin (100 I*·*U/50 *μ*g/mL) (Sigma Chemical Co., Saint Louis, MO, USA) in a humidified atmosphere containing 5% CO_2_ in air at 37°C.

### 2.3. Trypan Blue Exclusion Assay

A trypan blue exclusion assay was used to assess the effect of [6]-shogaol on B16F10 cell growth and viability. Briefly, following treatment with [6]-shogaol, cells were trypsinized and pelleted by centrifugation, and the cell pellet was resuspended in 300 *μ*L of DMEM media. Trypan blue (0.4% in PBS, 10 *μ*L) was added to a smaller aliquot (20 *μ*L) of cell suspension, and the cell number (viable unstained and nonviable blue) was counted using a hemocytometer under the microscope. Each sample was counted in triplicate, and each experiment was repeated at least three times.

### 2.4. Tyrosinase Activity Assay

The B16F10 cellular tyrosinase activity was determined according to a previously described method [23]. The cells were treated with *α*-MSH (100 nM) for 24 h and then further treated with various concentrations of [6]-shogaol (1, 5, 10, and 20 mg/mL) or arbutin (2.0 mM) for another 24 h. After these treatments, the cells were washed twice with PBS and homogenized with 50 mM PBS (pH 7.5) buffer containing 1.0% Triton X-100 and 0.1 mM PMSF (phenylmethylsulfonyl fluoride; a serine proteinase inhibitor). Intracellular tyrosinase activity was monitored as follows. The cellular extracts (100 *μ*L) were mixed with freshly prepared L-DOPA solution (0.1% in phosphate-buffered saline) and incubated at 37°C for 30 min. The absorbance at 490 nm was measured with a Gen 5 microplate reader (BIO-TEK Instrument, Winooski, VT) to monitor the production of dopachrome.

### 2.5. Melanin Content Measurement

The B16F10 cells were first stimulated with *α*-MSH (100 nM) for 24 h and then further treated with chemical inhibitors (10 *μ*M) or combined with [6]-shogaol for an additional 24 h. After the treatments, the cells were detached by incubation in trypsin/EDTA and subsequently centrifuged at 5,000 g for 5 min. The cell pellets were then solubilized in 1 N NaOH at 60°C for 60 min. The melanin content was assayed by spectrophotometric analysis at 405 nm absorbance [24].

### 2.6. Western Blot Analysis

Cells were lysed in PBS containing 1% Nonidet P-40, 0.5% sodium deoxycholate, 0.1% sodium dodecyl sulfate (SDS), 5 *μ*g/mL aprotinin, 100 *μ*g/mL phenylmethylsulfonyl fluoride, 1 *μ*g/mL pepstatin A, and 1 mM ethylenediaminetetraacetic acid (EDTA) at 4°C for 20 min. Total lysates were quantified using a microBCA kit (Thermo Fisher Scientific, Rockford, IL). Proteins (20 *μ*g) were resolved by SDS-polyacrylamide gel electrophoresis and electrophoretically transferred to a PVDF membrane. The membrane was blocked in 5% fat-free milk in PBST buffer (PBS with 0.05% Tween-20) followed by incubation overnight with the following primary antibodies diluted in PBST buffer: rabbit anti-mouse MITF antibody (1 : 1000), rabbit anti-mouse TRP1 antibody (1 : 2000), rabbit anti-mouse TRP2 antibody (1 : 1000), rabbit anti-mouse AKT antibody (1 : 1000), rabbit anti-mouse p-AKT antibody (1 : 1000), rabbit anti-mouse *β*-actin antibody (1 : 10,000), rabbit anti-mouse ERK antibody (1 : 1000), rabbit anti-mouse p-ERK antibody (1 : 1000) (Santa Cruz Biotech, Dallas, TX), and rabbit anti-mouse tyrosinase antibody (1 : 3000) (Epitomics, Burlingame, CA). The primary antibodies were removed, and the membrane was washed extensively in PBST buffer. Subsequent incubation with horseradish peroxidase-conjugated goat anti-rabbit antibodies (1 : 20000, Santa Cruz Biotech, Dallas, TX) was performed at room temperature for 2 h. The membrane was washed extensively in PBST buffer to remove any excess secondary antibodies, and the blot was visualized with enhanced chemiluminescence reagent (GE Healthcare, Piscataway, NJ).

### 2.7. Two-Step RT-PCR

RNA samples were reverse-transcribed for 120 min at 37°C with High Capacity cDNA Reverse Transcription Kit according to the standard protocol detailed by the supplier (Applied Biosystems). Quantitative PCR was performed as follows: 10 min at 95°C, 40 cycles of 15 sec each at 95°C, 1 min at 60°C using 2x Power SYBR Green PCR Master Mix (Applied Biosystems), and 200 nM of forward and reverse primers. Each assay was run on an Applied Biosystems 7300 Real-Time PCR system in triplicate and expression fold-changes were determined using the comparative CT method. Relative quantification was performed using GAPDH as an endogenous control.

### 2.8. Immunofluorescence Staining

B16F10 cells were fixed with 4% paraformaldehyde in 250 mM Hepes, pH 7.4, freshly diluted from 16% stocks stored at –20°C. After 5 min at room temperature, the cells were washed with phosphate-buffered saline and treated for 1 h at 4°C with blocking solution (PBST, 1% FBS). Gels were incubated with primary antibodies in blocking solution overnight at 4°C in a wet chamber. After washing in PBST, the cells were incubated with appropriate secondary antibody mixtures in blocking solution for at least 1 h at room temperature. The cells were washed three times in PBS and mounted in Gold antifade reagent with DAPI (Life Technologies, Carlsbad, CA). Confocal analysis was performed using a Leica TCS SP2 confocal microscope: 10 horizontal scans using a 63x (1.3 NA) oil immersion objective were recorded for each image with the imaging software (exported as a TIFF file).

### 2.9. Statistical Analysis

The statistical significance of differences was evaluated by ANOVA test after examining the variances; *P* < 0.05 (marked as “ *”), *P* < 0.01 (marked as “ **”), or *P* < 0.001 (marked as “ ***”) was considered to be statistically significant.

## 3. Results

**Figure 2:**
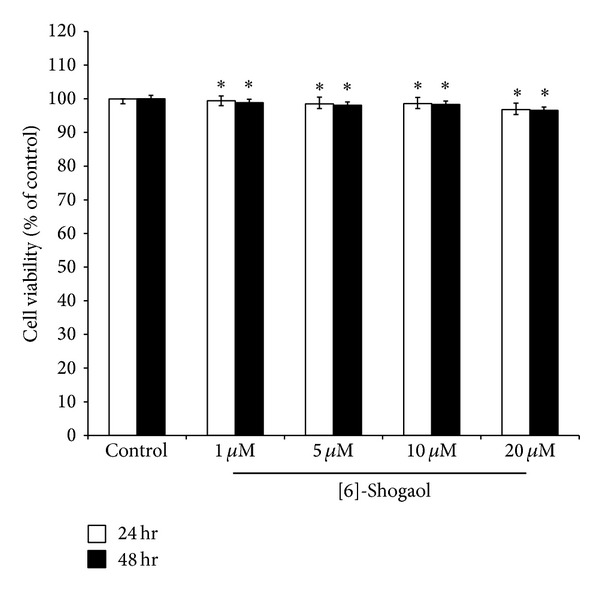
Effect of [6]-shogaol on the proliferation of B16F10 cells. Cell viability was measured by trypan blue dye exclusion method after 24 h incubation. Data are expressed as a percentage of the number of viable cells observed with the control, and each column is presented as the mean ± SD from two or three independent experiments performed in triplicate. *P < 0.05 compared to control by ANOVA test.

The cells treated with various concentrations of [6]-shogaol (1, 5, 10, or 20 *μ*M) for 24 h or 48 h were evaluated for cell viability based on the trypan blue exclusion assay. The results are expressed as percent viability relative to untreated control (Figure 2). The different concentrations of [6]-shogaol exhibited noncytotoxic effects on B16F10 cell viability.

When B16F10 cells were cultured in medium containing [6]-shogaol, the stimulation of tyrosinase activity by *α*-MSH was reduced to 87%, 68%, 58%, and 43% by 1, 5, 10, and 20 *μ*M [6]-shogaol, respectively. The IC_50_ of [6]-shogaol was 15.75 *μ*M. In addition, the intracellular tyrosinase activity was 64% in the arbutin-treated cells (Table 1). Thus, [6]-shogaol acts as a potential tyrosinase inhibitor. Additionally, the melanin content induced by *α*-MSH was decreased to 72% by 20 *μ*M [6]-shogaol. The residual melanin content in arbutin-treated cells was 66% of control. We also investigated the dependence of the inhibitory effect of [6]-shogaol on the ERK and PI3K signaling pathways. Cells pretreated with *α*-MSH were further treated with MEK (U0126), ERK (PD98059), or PI3K (LY294002) inhibitors with [6]-shogaol (Table 1). Cells added with MEK or ERK signal inhibitors exhibited increased melanin content compared to cells treated only with [6]-shogaol. The same pattern was evident using LY294002. The results showed that [6]-shogaol reduced the melanogenic activity of *α*-MSH-stimulated B16F10 cells through ERK and PI3K signaling, which inhibits intracellular tyrosinase activity and subsequently decreases melanin production.

**Figure 3:**
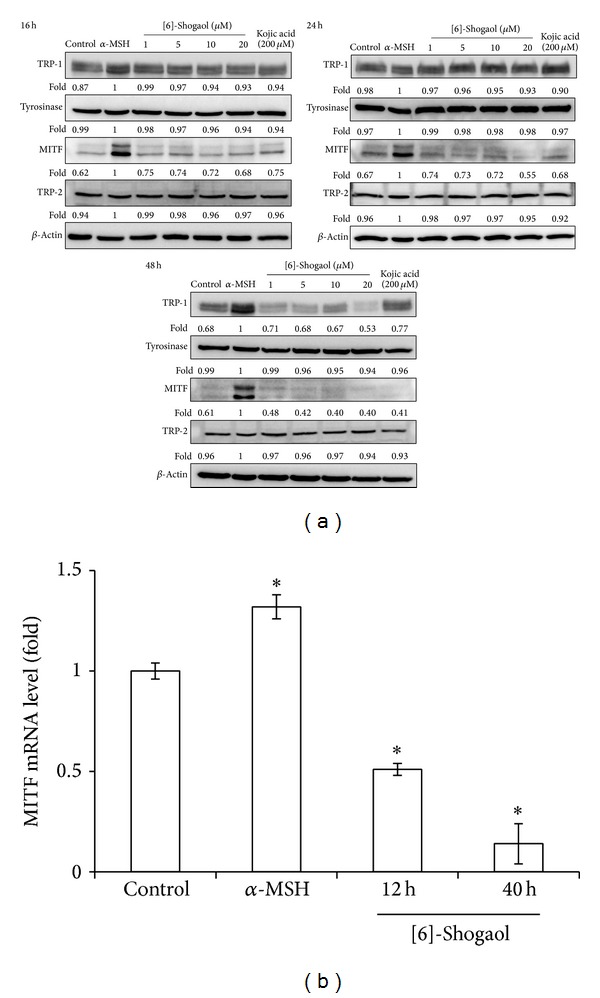
[6]-Shogaol inhibits melanogenic regulatory protein expression in B16F10 cells. (a) The tyrosinase, TRP-1, TRP-2, and MITF levels were determined by western blotting and *β*-actin was used as loading control. Relative expression levels were compared to *α*-MSH-treated cells and presented as the mean ± SD for three independent experiments performed in triplicate. (b) Total RNA from [6]-shogaol stimulated B16F10 cells at the indicated time was collected. MITF mRNA levels were examined by real-time RT-PCR using GAPDH as an internal control (*P < 0.05 versus control).

To elucidate the mechanism of the influence of [6]-shogaol on melanogenesis, the expression of tyrosinase, TRP1, TRP2, and MITF in *α*-MSH-stimulated B16F10 cells was analyzed by western blotting. B16F10 cells were incubated with 100 nM of *α*-MSH and then exposed to the [6]-shogaol at the specified time points. [6]-Shogaol inhibited *α*-MSH-induced MITF expression to 0.68-, 0.55-, and 0.4-fold of control at 16 h, 24 h, and 48 h, respectively (Figure 3(a)). The inhibitory effects of [6]-shogaol on MITF production are similar to the effects of 200 *μ*M of kojic acid (KA), which is an established effective melanogenesis inhibitor [25]. This inhibition was correlated with the downregulation of TRP-1 expression by 0.53-fold at 48 h. In contrast, [6]-shogaol treatment did not display changes in the tyrosinase and TRP2 levels in *α*-MSH-treated B16F10 cells. Decreased MITF mRNA levels were also detected in the presence of 20 *μ*M [6]-shogaol (Figure 3(b)).

**Figure 4:**
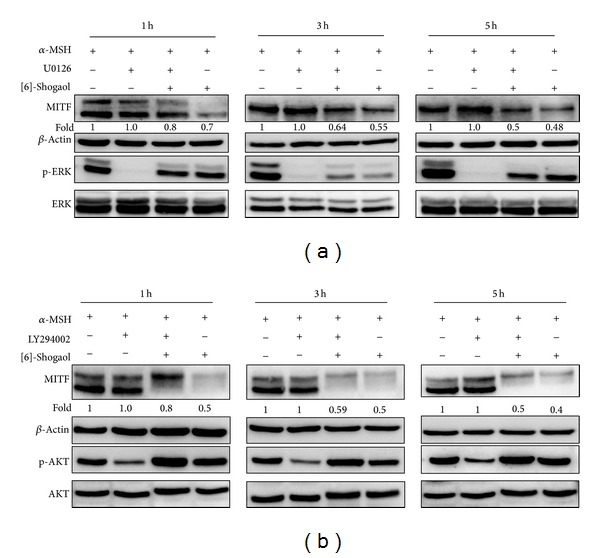
Effects of [6]-shogaol on the phosphorylation of ERK and Akt and on the MITF in B16F10 in the presence or absence of U0126 (a) or LY294002 (b). The *α*-MSH incubated B16F10 cells pretreated with U0126 were stimulated with [6]-shogaol for 72 h. The expression levels of the MITF, pERK, ERK, pAkt, and Akt were analysed by western blot using specific antibodies. The data represent at least three independent experiments. Relative density ratios of MITF over *β*-actin were shown as the mean values compared with *α*-MSH-treated cells.

To further confirm the role of ERK and PI3K signaling pathways in [6]-shogaol-induced inhibition of melanogenesis, three kinase inhibitors were incubated before they were exposed to [6]-shogaol. Cells pretreated with LY294002 or U0126 before stimulation with [6]-shogaol displayed increased expression of MITF compared to cells treated only with [6]-shogaol by increasing AKT or ERK phosphorylation, respectively (Figure 4). The MITF expression in B16F10 cells cotreated with *α*-MSH and PD98059 was higher than in the cells treated with *α*-MSH alone. However, the synergistic effect of *α*-MSH and PD98059 on the MITF was decreased by the [6]-shogaol treatment. These results suggested that the [6]-shogaol-induced antimelanogenic effect may be mediated by activation of the AKT and ERK pathway. In addition, the protein expression levels of p-ERK and p-AKT were also increased by [6]-shogaol (Figure 4). Furthermore, blocking p-ERK and PI3K increased the melanin content attenuated by [6]-shogaol (Table 1). These results demonstrate that the MITF inhibitory effect of [6]-shogaol is dependent on the ERK and PI3K signaling pathways.

**Figure 5:**
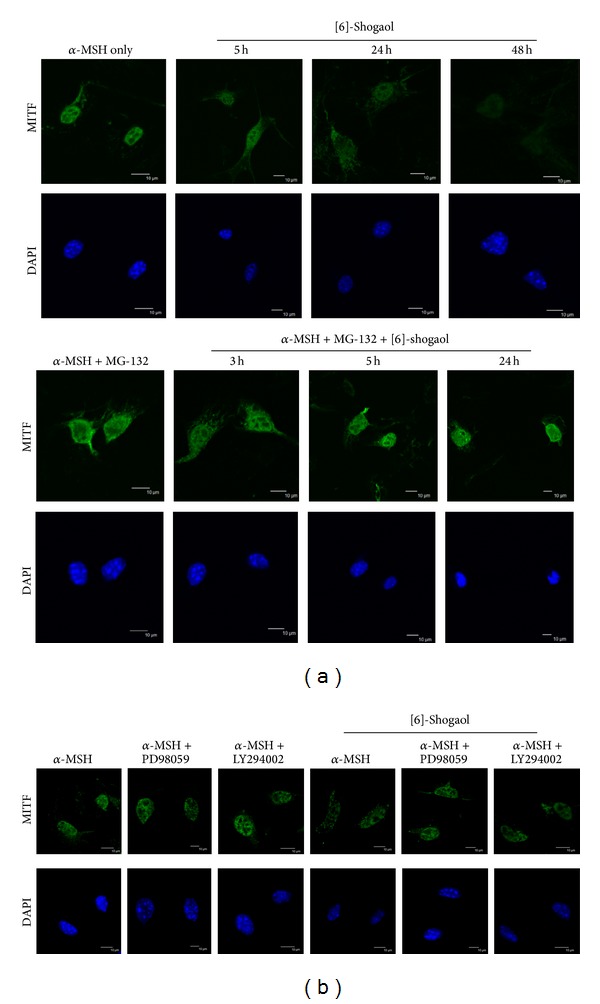
Immunofluorescence analysis. B16F10 cells were treated with [6]-shogaol and fixed at the indicated time and subjected to immunofluorescence detection of MITF protein. DAPI staining was used to illustrate the nuclei. Immunolocalization of MITF in B16F10 cells. The results from three independent experiments are presented. Bars: 10 *μ*m.

The downregulation of MITF expressions by [6]-shogaol was also investigated by immunofluorescence staining assay. As shown in Figure 5, the fluorescence signal for MITF was mainly observed in the nuclei of B16F10 cells. Immunofluorescent staining showed a substantial decrease in overall MITF staining after 24 h of incubation with [6]-shogaol; this staining was absent in the nucleus and showed a diffuse cytosolic distribution after [6]-shogaol treatment. Downregulation of MITF was prevented by pretreatment with proteasome inhibitor MG-132, suggesting the involvement of the proteasome in the downregulation of MITF by [6]-shogaol. We also used specific inhibitors, U0126 and PD98059, which were able to reverse the MITF downregulation induced by [6]-shogaol (Figure 5(b)). The results clearly indicate that activated ERK and PI3K are necessary to evoke the effects observed.

## 4. Discussion

A wide variety of phenyl alkyl ketone compounds derived from natural products possess potent antioxidant and antimelanogenic activities [26, 27]. This study examined the effects of [6]-shogaol on the melanogenesis signaling pathway activated by *α*-MSH. In this study, we investigated the effect of [6]-shogaol on tyrosinase activity. [6]-Shogaol significantly inhibits tyrosinase activity in a dose-dependent manner. This inhibitory effect of [6]-shogaol was stronger than that of arbutin. The nonlinear relationship between intracellular tyrosinase activities and melanin content, which may be due not only to different amounts of tyrosinase present in the melanocytes but also possibly to differences in the catalytic activities of tyrosinase in the cells, was found. These results suggest that the decrease in melanogenesis induced by [6]-shogaol could be achieved via its inhibitory action on the signaling pathway that regulates tyrosinase activity.

The activation of ERK signaling downregulates melanogenesis by inhibiting MITF activity [28]. Activation of ERKs leads to the phosphorylation of Ser73 of MITF, and, together with recruitment of the transcriptional coactivator, p300, this process targets MITF for ubiquitination and proteasome-mediated degradation [16]. In this study, [6]-shogaol clearly stimulated the phosphorylation of ERK and inhibited the synthesis of melanin. Therefore, the activation of the ERK signaling pathway by [6]-shogaol might play an important role in the depigmenting effects. In addition, activation of the AKT signaling pathway plays a key role in inhibiting melanogenesis [29]. Previous studies have also shown that the activation of AKT suppressed melanin production in human melanoma G631 and murine melanocyte Melan-A cells [30]. Inhibition of the AKT pathway by the AKT inhibitor, LY294002, increased melanin synthesis in B16F10 cells [31]. Thus, [6]-shogaol is an effective suppressor of hyperpigmentation caused by *α*-MSH via ERK and AKT activation and by the subsequent downregulation of MITF and TRP-1 production. In 2012, Yao et al. demonstrated that [6]-shogaol inhibited MITF protein expression via the ERK signaling pathway but not AKT activation in B16F10 cells. In their study, the antimelanogenic activity of [6]-shogaol was observed using 1, 5, or 10 *μ*M [6]-shogaol on B16F10 cells in the absence of *α*-MSH. Further, the authors did not determine the gene expression level of MITF [32]. By comparison, *α*-MSH prestimulation of B16F10 cells was performed in our study to demonstrate the antimelanogenic effect of [6]-shogaol. We next found that inhibitors of ERK or PI3K do in fact block [6]-shogaol-inhibited melanin synthesis and tyrosinase activity in B16F10 cells. Thus, [6]-shogaol-induced inhibition of melanogenesis occurs through phosphorylation of ERK and AKT. These differences in the study findings may suggest that [6]-shogaol inhibits basal level of pigment production and various stimuli that lead to the production of *α*-MSH and the subsequent melanin production. The inhibitory effects of  [6]-shogaol on melanin synthesis and phosphorylation of ERK demonstrated in our study are in line with the above mentioned role of ERK signaling pathway in pigment formation. The antimelanogenic mechanism of [6]-shogaol was through ERK, or both the ERK and AKT pathways, in the presence of *α*-MSH, respectively.

The present study showed that [6]-shogaol reduced MITF protein levels and changed MITF mRNA expression. Moreover, the proteasomal inhibitor, MG132, almost completely recovered MITF levels from the downregulation by [6]-shogaol. This indicates that MITF was also proteolytic degraded by [6]-shogaol. Here we also show that the ERK and AKT signaling pathways are both involved in the transactivation and stability of MITF [33]. ERK and AKT inhibitors attenuated the inhibition of MITF degradation by [6]-shogaol. The data showed that [6]-shogaol downregulated MITF protein expression at the level of transcript synthesis as well as protein degradation. Therefore, we propose that [6]-shogaol may be useful as an antimelanogenesis agent.

**Figure 6:**
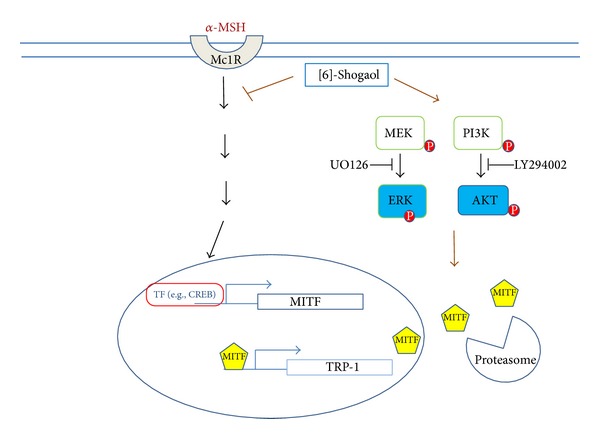
The proposed mechanism by which [6]-shogaol inhibits melanin biosynthesis. Cartoon shows that [6]-shogaol decreases expression of MITF in B16 Cells. The suppressive effect of [6]-shogaol on melanin synthesis was induced through ERK and Akt activation. Suppression of the ERK or AKT pathway diminishes [6]-shogaol-induced inhibition of melanin synthesis. Moreover, [6]-shogaol inhibits melanin synthesis via proteasomal degradation of MITF.

We observed that [6]-shogaol suppressed the expression levels of MITF and TRP-1 but did not decrease the synthesis of tyrosinase and TRP-2. This difference may be because MITF is required but not sufficient to induce the expression of melanogenic genes. It is plausible that different genes are targeted in distinct contexts and that the transcriptional activity of MITF may be tightly regulated in a signal-dependent fashion [8, 9, 34]. [6]-Shogaol inhibits the activity of tyrosinase, reduces the cellular melanin content, phosphorylates ERKs and AKT, and inhibits the expression of MITF and TRP1 in *α*-MSH-stimulated B16F10 cells. These results suggested that [6]-shogaol inhibits melanogenesis signaling by activating the ERK or AKT signal pathway-mediated suppression of MITF and its downstream molecular targets, tyrosinase and TRP1 (Figure 6).

## Supplementary Material

The supplement materials for Table 1: contain the raw data for inhibition percentage of tyrosinase activity by [6]-Shogaol and melanin content in the presence of [6]-Shogaol alone or combined with speific kinase inhibitors such as U0126, PD98059 and LY294002. Also, the supplement figure 2 exhibted [6]-Shogaol inhibit MITF expression in the absence of *α*-MSH.

## Figures and Tables

**Table 1:** Effect of [6]-shogaol on *α*-MSH-induced tyrosinase activity and melanin synthesis in B16F10 cells.

	Tyrosinase activity (% of *α*-MSH control)					
[6]-Shogaol (*μ*M)	1	5	10	15	20	Arbutin (2 mM)
	87.91^a^	68.19^b^	58.03^b^	51.99^b^	43.68^b^	64.26^b^

	Melanin content (% of *α*-MSH control)					
Inhibitors 20 *μ*M/ [6]-shogaol 20 *μ*M	[6]-Shogaol	U0126 [6]-shogaol		PD98059 [6]-shogaol	LY294002 [6]-shogaol	Arbutin (2 mM)
	72.13^b^	95.21^b^		97.43^b^	102.05^b^	66.06^b^

^a^ *P* < 0.05; ^b^ *P* < 0.001.
