# Supplementary material for: [6]-Shogaol Inhibits α-MSH-Induced Melanogenesis through the Acceleration of ERK and PI3K/Akt-Mediated MITF Degradation
Source: Biomed Res Int. 2014 Jun 19;2014:842569. doi: 10.1155/2014/842569 (PMC4090493; doi:10.1155/2014/842569)

Manuscript ID: 2738/842569.v2

Title: [6]-Shogaol Inhibits α-MSH induced Melanogenesis through the Acceleration of ERK and PI3K/Akt -Mediated MITF Degradation

Authors: Huey-Chun Huang, Shu-Jen Chang, Chia-Yin Wu, Hui-Ju Ke, Tsong-Min Chang^*^

Supplement data:

Supplement for Table 1

| [6]-Shogaol | Tyrosinase activity(%) | Tyrosinase activity(%) | Tyrosinase activity(%) | average | SD |  |
| --- | --- | --- | --- | --- | --- | --- |
| 1 μM | 87.65 | 88.12 | 87.97 | 87.913333 | 0.196015872 |  |
| 5 μM | 67.78 | 68.81 | 67.98 | 68.19 | 0.44594469 |  |
| 10 μM | 57.73 | 58.24 | 58.12 | 58.03 | 0.217715411 |  |
| 15 μM | 52.01 | 52.03 | 51.94 | 51.993333 | 0.038586123 |  |
| 20 μM | 43.67 | 43.73 | 43.64 | 43.68 | 0.037416574 |  |
| Arbutin(2 mM) | 64.12 | 64.48 | 64.17 | 64.256667 | 0.159234279 |  |
|  |  |  |  |  |  |  |
|  |  |  |  |  |  |  |
|  | Melanin content(%) | Melanin content(%) | Melanin content(%) | average | SD |  |
| [6]-Shogaol (20 μM) | 71.96 | 72.41 | 72.02 | 72.13 | 0.199499373 |  |
| [6]-Shogaol + U0126 | 95.43 | 95.18 | 95.02 | 95.21 | 0.168720676 |  |
| [6]-Shogaol + PD98059 | 97.37 | 98.01 | 96.91 | 97.43 | 0.451072795 |  |
| [6]−Shogaol + LY294002 | 101.81 | 102.03 | 102.32 | 102.05333 | 0.208859336 |  |
| Arbutin(2 mM) | 66.34 | 65.73 | 66.12 | 66.063333 | 0.252234459 |  |
|  |  |  |  |  |  |  |
|  |  |  |  |  |  |  |

Supplement figure 2


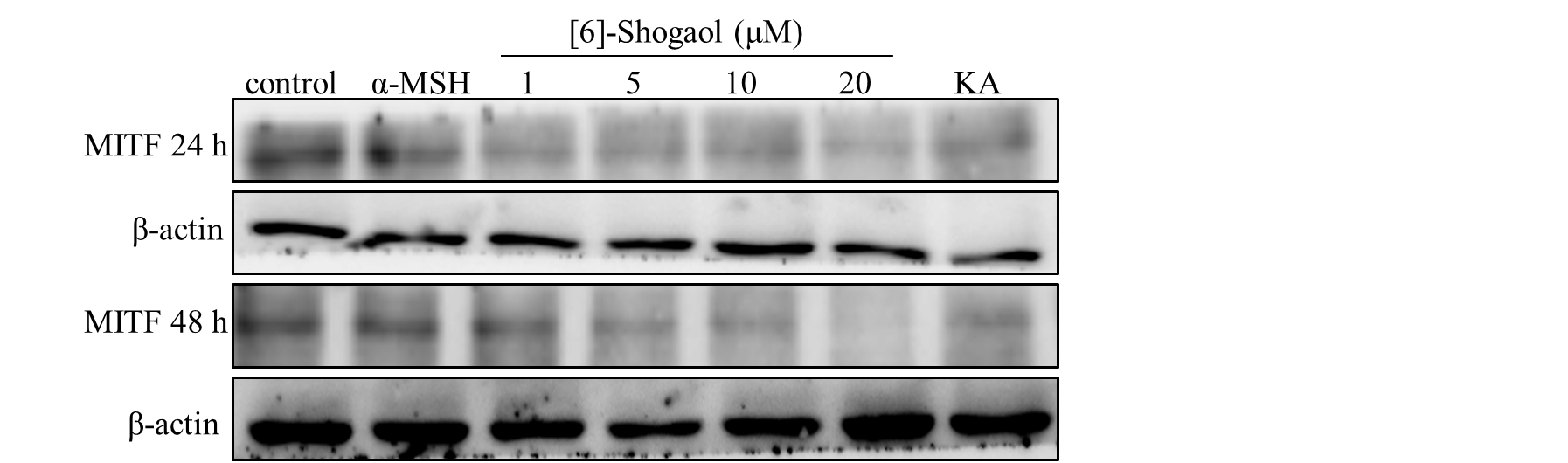

Supplement: Supplementary file 1 — The supplement materials for Table 1: contain the raw data for inhibition percentage of tyrosinase activity by [6]-Shogaol and melanin content in the presence of [6]-Shogaol alone or combined with speific kinase inhibitors such as U0126, PD98059 and LY294002. Also, the supplement figure 2 exhibted [6]-Shogaol inhibit MITF expression in the absence of α-MSH. [file 842569.f1.docx]
